# Felon

**Published:** 1872-04

**Authors:** 


### FELON.

A felon is a contemptible fellow, one that preys upon our lives and property, and is only safe when confined. Not so, however, with the one known also as *whitlow*, and *paronychia;* he is a different sort of a chap, and the sooner you let him free the better. Very few persons among the laity, we find, know what a felon is. It is regarded by many as some sort of foreign substance—a something that can be taken hold of and removed. We have been greatly surprised to find the following item going the rounds of the daily press, and in some instances have encountered it in medical journals, without its authenticity even being challenged:

"The *London Lancet* says as soon as the pulsation which indicates the disease is felt, put directly on a fly blister about the size of your thumb nail, and let it remain for six hours, at the expiration of which time, directly under the surface of the blister may be seen the felon, which can be instantly taken out with the point of a needle or a lancet."

If any of our readers have read this nonsensical cure for felon, and have employed it, we should be glad to learn from them what a felon looks like when taken upon the point of a lancet or needle; would be delighted to know its color, shape, and consistence.

The truth is, a felon is an inflammation of the covering membrane of the bone, (the *periosteum*) or tendon, or both, and is then termed by physicians the "deep seated" variety, in contradistinction to the superficial sort, which attack the skin and cellular substance, immediately beneath.

The superficial felon usually exhibits itself immediately about, or beneath the nail, without much if any swelling, the finger appearing slightly red, at the end, and is excrutiatingly painful, keeping up a constant throbbing for two or three days, when matter shows itself just beneath the skin.

The deep seated variety are the most painful and dangerous, often causing complete destruction of the finger, and not infrequently of the hand. The pain is of the most intense, throbbing character, depriving the patient of sleep for days and even weeks. The inflammation extends to the hand, thence to the elbow, and even to the shoulder, causing more or less constitutional disturbance. If the inflammation is not speedily checked, the swelling becomes enormous, resembling malignant erysipelas, and should the matter not be allowed to escape, will burrow beneath the integuments, requiring lancing of the hand, wrist, and often the arm. Frequently gangrene or mortification occurs, in which the finger, if not the hand, is sacrificed.

There is but one safe treatment for felons:

Cut freely and largely, down to the bone. Not a small cut, just large enough to allow the matter to escape, *but lay the finger open its entire length*, if diseased near its centre, and from the second joint, if at the end. The cutting must be done early, particularly if a deep seated felon. Do not wait for the matter to form, for there is no room for it, beneath the heavy tendons of the finger, and its presence there will soon cause most horrible pain from pressure. *Open the finger the moment you are satisfied that you have a felon*; cutting clear through the periosteum, to the bone; do not wait for matter to form. In this manner you not only prevent the matter from burrowing beneath the tendon, but very often arrest the further progress of the disease. After the finger has been laid open freely, then poultice with hot bread-and-milk, over which laudanum may be poured. Change the poultice frequently, keeping it as hot as can be borne. Be careful to have *enough* poultice about the finger—let it be completely imbedded with a soft, smooth one, an inch or more in thickness.

If our instructions are closely followed—the finger being opened immediately and freely—we will see no more deformed fingers, nor crippled hands. We regard this procedure as the only cure for genuine felon.

